# Exploration of Cutting Processing Mode of Low-Rigidity Parts for Intelligent Manufacturing

**DOI:** 10.3390/mi16060624

**Published:** 2025-05-26

**Authors:** Jianping Zhu, Xinna Liu, Hui Peng, Wei Liu, Zhiyong Li

**Affiliations:** 1 Rongcheng College, Harbin University of Science and Technology, Weihai 264800, China; zhujianping2017@126.com (J.Z.); woshipenghui@126.com (H.P.); 2 Rongcheng Kangyi New Material Technology Co., Ltd., Weihai 264800, China; 3 Mechanical Engineering College, Shandong University of Technology, Zibo 255049, China; lzy761012@sdut.edu.cn

**Keywords:** intelligent manufacturing, low-rigidity parts, cutting mode, deep learning, big data, state awareness

## Abstract

With the development of intelligent manufacturing technology, the manufacturing industry is gradually realizing intelligent production. Especially for metal cutting with extremely complex processes, it is of great significance to realize intelligence. Taking the cutting process of aero-engine typical low-rigidity parts as the main line, this article builds an intelligent processing architecture based on a big data platform, which includes customized design of cutting tools, intelligent optimization of cutting parameters, simulation of cutting conditions, and online monitoring and control of cutting processes. At the same time, the realization of related key technologies is explained. Then, this article introduces in detail the intelligent decision-making process based on deep learning, the customized tool design process based on structural features, the simulation process of cutting based on geometric features of parts, as well as the monitoring and control process of Numerical Control (NC) machining based on condition perception. In addition, based on the processing requirements and difficulties of specific parts, formulate a specific intelligent implementation plan under this processing mode. Through the implementation of the above architecture and key technologies, the cutting processing system can automatically optimize the cutting parameters according to real-time working conditions and adjust its own cutting conditions. At the same time, machine tool condition, cutting tool condition, and low-rigidity part condition are real-time monitored to achieve high-precision, efficient, intelligent, and precise cutting of low-rigidity parts. The proposed architecture can provide a reference model for the research and application of intelligent cutting technology for low-rigidity parts.

## 1. Introduction

In recent years, the target pursued by the manufacturing market has experienced changes in large-scale production, low-cost manufacturing, product quality, market response speed, knowledge, and service, and is now gradually transforming from automation and information technology to intelligence [1]. With the rapid development of information technology, such as computer technology, sensing technology, and communication technology, artificial intelligence, big data, Internet of Things, and cloud computing have initially obtained supporting conditions [2,3]. China and major developed countries have begun to set up various research programs, including “Made in China 2025” and “Industry 4.0”, to support the research of intelligent manufacturing technology. Some famous companies in the world, such as Siemens and Sandvik, have been promoting the construction of intelligent factories, and academic issues related to intelligent manufacturing technology have become a research hotspot. Therefore, it has become an inevitable trend for the development of the manufacturing industry to transform and upgrade to intelligent manufacturing, identify and predict dynamic market demand quickly and accurately, integrate it into the manufacturing process of products, and build a dynamic data-driven closed-loop manufacturing model [4,5].

In the manufacturing process of typical thin-walled key parts (low-rigidity parts) for aeroengines, cutting is the main means. Generally based on Computer Numerical Control (CNC) machine tools, CNC workshops are established according to product types or process methods, and some local digital production lines and digital workshops are formed through information construction and digital engineering implementation. However, for the manufacturing site status and environmental information, key process equipment, status process monitoring, real-time control, and other links are still lacking systematic intelligent processing capabilities, resulting in the information processing capacity of the entire manufacturing process lagging. Therefore, it is necessary to conduct in-depth research and improvement on the systematic intelligent processing ability of information in the field of metal cutting and processing, and the digital, real-time, and accurate execution ability of the processing process.

Low-rigidity parts such as integral wall panel, frame, integral wing rib, and turbine blade of the engine have the characteristics of complex structure, thin wall, weak stiffness, and high surface accuracy requirements (Ra < 0.8 μm). In the machining process, the metal removal rate is large, which is prone to nonlinear and strong time-varying problems accompanied by material removal, resulting in the redistribution of spatial stress and the evolution of structural stiffness, leading to great deformation. The elastic recovery will occur after the deformation of the cutter, and then the phenomenon of letting the cutter appear, making the actual milling width not equal to the nominal value. At the same time, with the continuous removal of materials, the release of residual stress and the re-equilibrium state will affect the deformation of parts and ultimately lead to the reduction of product processing accuracy, making it difficult to ensure the processing quality [6]. Due to the small elastic modulus (such as: the elastic modulus of TC4 is approximately 110 GPa) of low-rigidity parts, poor thermal conductivity, and small tool contact area, the tool is constantly subjected to thermal coupling, which makes the tool wear seriously in the cutting process, and even causes the tool fracture and failure, resulting in the flutter and damage of the machine tool and the workpiece. Moreover, the material cost of such parts is high, the processing time is long and does not allow scrapping, so it is of great significance to monitor the state of the machine tool, tool and workpiece in the cutting process in real time and realize the monitoring and control of the CNC machining of such parts.

In the process of metal cutting, CNC machine tools play a crucial role in product manufacturing. At present, the processing of such parts is based on the geometric shape of the part and the given cutting parameters to generate a CNC machining program for processing. However, with the rapid development of artificial intelligence, only the feed rate and spindle speed can be adjusted, and information about the workpiece, cutting tool, processing features, and processing parameters cannot be obtained. CNC machining can no longer meet the current processing mode. The cutting process of very complex machine tools, tools, and workpiece state changes into the processing process should be considered. Even if the cutting force, cutting temperature, spindle current, and acoustic monitoring can be monitored in real time, it can only warn, and it is still necessary to manually observe and analyze the state of the machine tool, workpiece, and tools, and adjust the cutting parameters according to experience. Many scholars have conducted research on the intelligence of CNC machine tools [7,8,9]. Therefore, it is necessary to conduct in-depth research on the intelligent processing ability and real-time execution ability of the cutting process information of this kind of part.

With the rapid development of cloud computing, big data, Internet of Things, artificial intelligence, and other technologies, cyber-physical systems (CPS) are promoting intelligent manufacturing technology to gradually realize deep intelligence. Artificial intelligence is the control center, the Internet of Things is the collection end of big data and the execution end of artificial intelligence, through the Internet of things to automatically collect all kinds of data information, so as to obtain big data, when the data has accumulated to a certain extent, it needs a platform for intelligent processing and analysis of big data (cloud computing) for storage and memory, so as to achieve deep artificial intelligence based on big data. Through the artificial intelligence technology based on cloud computing and big data and the Internet of Things technology, an intelligent manufacturing system integrating computing, communication and control can be realized, so that the intelligent manufacturing system can realize state perception, real-time analysis, intelligent decision-making, and accurate execution to achieve visual monitoring of the product manufacturing process.

In 2006, HINTON et al. [10] discussed deep learning theory for the first time in Science, which started its wave in academia and industry. Deep learning uses big data to learn features and characterize the rich intrinsic information of the data to improve the accuracy of classification or prediction. At present, deep learning has made breakthroughs in big data analysis in areas such as speech recognition and image recognition. At the same time, the application of deep learning in the mechanical field is also increasing. For example, Lei Yaguo et al. [11] proposed a big data health monitoring method for mechanical equipment based on deep learning theory, which realized adaptive fault feature extraction and accurate health status identification for different fault locations and types of multistage gear transmission systems under various working conditions and a large number of samples. Li X et al. [12] proposed a novel deep learning method for rotating machinery fault diagnosis and obtained a diagnostic accuracy of up to 99.9%. There are also certain applications in the field of metal cutting, including tool selection [13], tool condition monitoring and prediction [14,15,16], and time-varying parameter processing [17], which have been well applied, but there are few applications in the field of aerospace typical parts cutting.

Based on the above research, this paper takes the cutting of typical low-rigidity parts of aerospace as the background, aiming at the problems such as high cutting cost, low cutting efficiency, short tool life, complex structure, thin wall and easy deformation, dimensional accuracy and surface quality difficulty to guarantee, combined with deep learning and big data technology. An intelligent manufacturing technology architecture for the cutting of low-rigidity parts is proposed, and the realization of its key technologies is explored, which provides a reference solution for the monitoring and control of the intelligent CNC machining of low-rigidity parts for aerospace.

## 2. Intelligent Manufacturing Technology Architecture for Cutting and Machining Low-Rigidity Parts

Metal cutting is a very complex process, involving physics, chemistry, mechanics, materials science, vibration, tribology, heat transfer, and other disciplines and fields related to knowledge and theory. Therefore, the intelligence of cutting process should collect and understand environmental information and self-information through sensing equipment and carry out self-learning and data mining through intelligent cooperation between humans and machines, intelligent processing and feedback regulation of manufacturing information, etc., so as to realize the analysis and judgment of machining process and plan the machine tool behavior [18].

**Figure 1:**
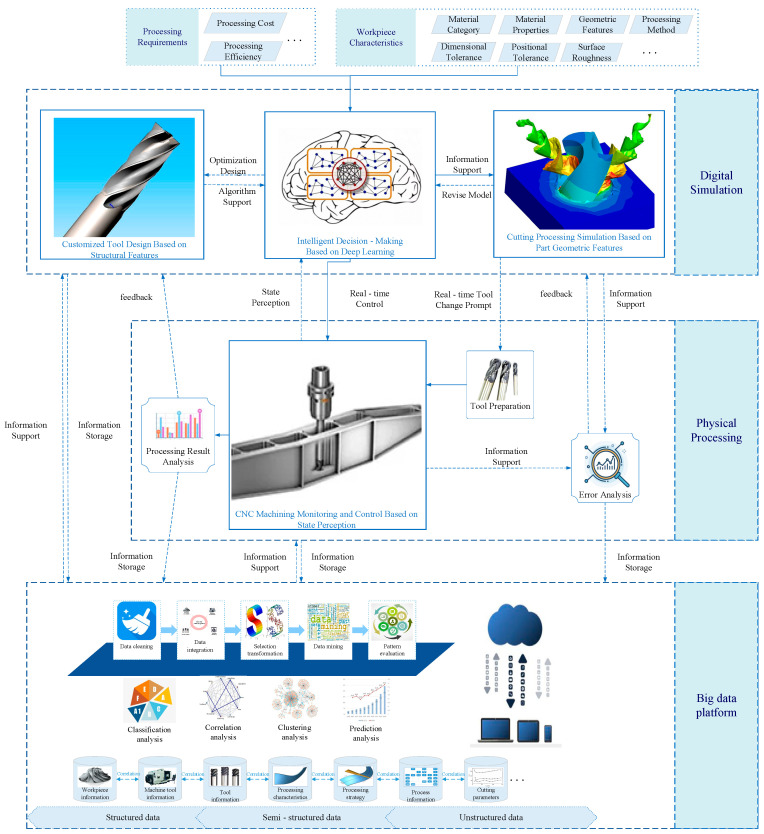
Intelligent manufacturing technology architecture for machining low-rigidity parts.

Aiming at the characteristics of high cutting cost, low cutting efficiency, short tool life, complex structure, thin wall, weak stiffness and high surface accuracy requirements for low-rigidity parts of aerospace engine, this paper proposes an intelligent manufacturing technology architecture for cutting, as shown in Figure 1, which includes three parts: digital simulation, physical processing and big data platform. Under the support of a big data platform, this architecture is a closed-loop intelligent manufacturing system composed of intelligent decision making based on deep learning, customized tool design based on features, cutting simulation based on geometric features of parts, and CNC machining monitoring and control based on state perception.

In Figure 1, the input of the whole system is the machining requirements such as tool cost and machining efficiency, as well as the workpiece characteristics and technical requirements such as material category, material characteristics, geometric characteristics, machining methods, dimensional tolerances, form and position tolerances, surface roughness, etc., and then the information exchange is carried out between each part.

(1) Digital simulation

Digital simulation includes three parts: custom tool design based on structural features, intelligent decision based on deep learning, and cutting simulation based on geometric features of parts.

➀ Custom tool design based on structural characteristics

Mainly because existing tools cannot meet the cutting requirements of special structural low-rigidity parts, custom-designed special tools are needed. Additionally, when the performance of existing tools is not ideal, optimizing their design can provide technical support for the future cutting of such parts.

➁ Intelligent decision making based on deep learning

This section is the core of the architecture. By training the algorithm model based on the cutting history information of low-rigidity parts provided by the big data platform, a deep learning model suitable for the machining conditions of low-rigidity parts is obtained, which provides top-level decision support for the intelligent processing of the architecture.

➂ Machining simulation based on geometric features of parts

This includes cutting process simulation and intelligent process planning based on the geometric features of parts. It enables the simulation and visualization of the cutting process, plans the cutting process tool path based on the geometric features of parts, automatically optimizes the process, and automatically generates CNC machining programs.

Digital simulation mainly includes two tasks:

- Before cutting parts

According to the characteristics and processing requirements of the workpiece to be machined, the digital simulation can intelligently match and make decisions on the machine tool information and tool information by using deep learning algorithms through the information support of the big data platform. When the existing tool processing cannot meet the requirements of the part processing or the matching degree is not ideal, special tools can be designed by custom tool design or optimized for existing tools. To ensure that the machine tools and tools can meet the requirements of parts processing, the information of the machine tools, tools, and parts is used as the input of the deep learning model to make intelligent decisions about cutting parameters and other information and provide information support for the simulation part.

The cutting process of low-rigidity parts is predicted and simulated by machining simulation based on the geometrical characteristics of parts, including cutting force, cutting temperature, cutting stress field and temperature field, and tool wear and chip shape simulation. By analyzing the simulation results, the tool design parameters and initial cutting parameters can be modified to some degree, and the data support for the cutting tool life prediction can be provided. At the same time, the simulation part can also obtain the real-time updated CNC machining program according to the intelligent decision of the part feature matching results, dynamic cutting parameters, and the data support of the big data platform, while the tool life is predicted and the tool replacement is prepared. Digital simulation through the simulation before the actual machining, and the problems that may arise in the machining process of low-rigidity parts can be found and solved in advance, and the dimensional accuracy and surface quality of parts, and even the residual stress, can be predicted.

b. In the process of cutting parts

Due to the existence of uncontrollable factors such as the site environment, there will be some differences between the measured data during the actual machining process and the simulated machining process data. The system will extract and process data features of all kinds of information according to real-time processing information of CNC machining monitoring and control feedback based on state perception, and the intelligent decision part will predict through each algorithm model suitable for the working condition, and obtain cutting parameter data for users to visualize, so as to obtain the current cutting processing state. The visualization of the machining process is realized through a simulation module. At the same time, compared with the simulation results corresponding to the time, the error analysis is carried out in real time, and the parameters of the simulation model are modified, and then the parameters of the algorithm are modified. At the same time, the real-time cutting parameters and numerical control program are optimized by the corresponding algorithm model, so as to achieve the purpose of guiding numerical control machining in real time through the cutting state perception, and realize the real-time monitoring and intelligent control of the machining process.

(2) Physical processing

After the machining accuracy and surface roughness of the predicted parts meet the requirements, the physical machining can be carried out. In the actual cutting process, the CNC machining monitoring and control part transmits the real-time cutting force information, cutting temperature information, cutting vibration information, acoustic emission information, current information measured by the force sensor, temperature sensor and other equipment to the real-time data layer of the intelligent decision-making part, and allows users to observe the changes of cutting parameters in real time through digital simulation. At the same time, the visualization of the cutting process is realized, allowing users to monitor the machine tool state, tool state, and workpiece state, and providing a real-time understanding of the processing state of the parts, and can change the cutting parameters or processing strategies at any time according to experience to achieve accurate machining.

Under the guidance of the intelligent decision part, real-time prediction of cutting tool wear and damage, forecasting the remaining life of the tool, and real-time display of the expected tool change time are possible, so that users can prepare the tool in advance. In addition, real-time reliability assessment is carried out based on big data, error analysis is carried out for differences in simulation results, analysis reports are output, and the accuracy of the digital simulation system is improved through self-learning.

When the machining of low-rigidity parts is completed, the output result analysis report, including whether the machining accuracy, surface roughness, etc., meets the expected requirements, and the analysis result information is fed back to the digital simulation to modify the intelligent decision model and the simulation model, expand the big data platform information, and provide information support for the future optimization design of the tool for the processing of low-rigidity parts with similar structural characteristics.

(3) Big data platform

The operation of digital simulation and physical processing is completed with the support of a big data platform. The platform contains all processing-related information of low-rigidity parts, such as workpiece information, tool information, machine tool information, process information, cutting parameters, processing characteristics, and processing strategies. Each instance library is integrated and linked, and there is a mapping relationship between each other.

**Figure 2:**
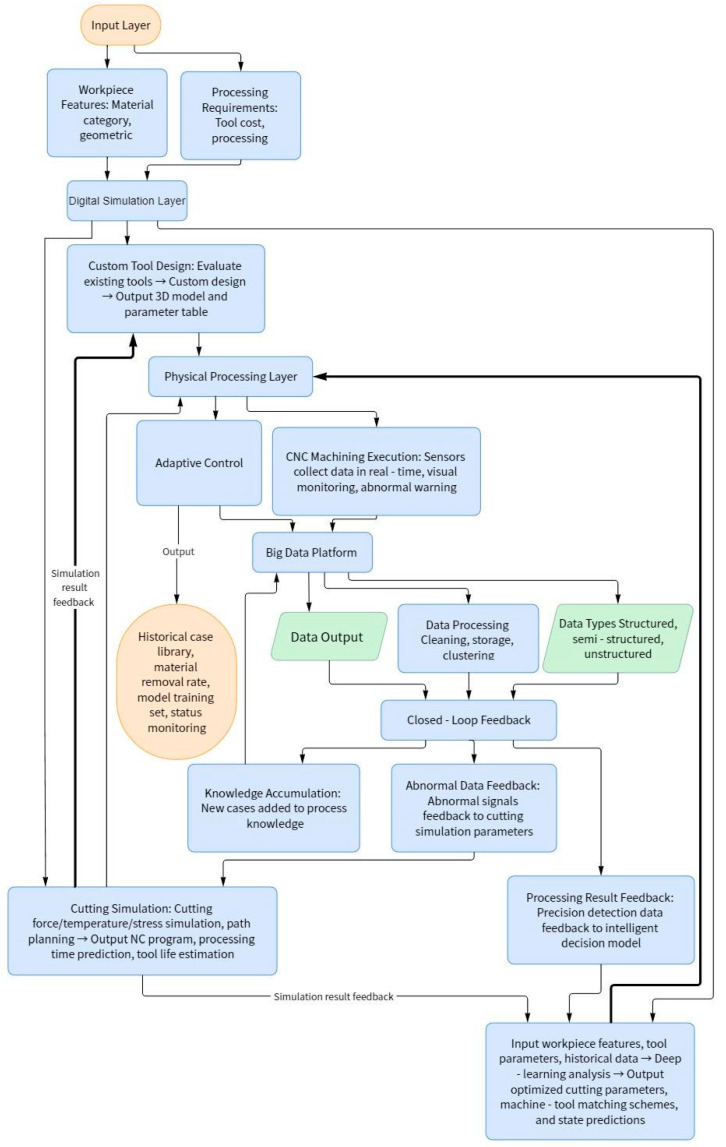
Intelligent manufacturing technology architecture for intelligent CNC machining of low-rigidity parts.

The information in the platform includes structured data with the same data type and same attribute values (such as tool information and machine tool information), semi-structured data with the same data type and different attribute values (such as geometric characteristics of low-rigidity parts), and unstructured data with no fixed structure (such as tool wear pictures). The above multi-source heterogeneous non-real-time data are processed using the Hadoop framework. For storage and management, real-time data are processed using the Storm framework. The data entering the platform are first cleaned to remove duplicate samples, abnormal samples, samples deviating from the overall distribution, etc. For missing values, complete, correct, and consistent data information can be stored in the data warehouse manually or by means of median, etc., so as to improve the correctness of data mining results. The multi-source heterogeneous data is logically or physically centralized to realize data sharing. By means of smooth aggregation, data generalization, normalization, and so on, the data is transformed into a form suitable for data mining. According to the data information in the data warehouse, appropriate analysis tools are selected; statistical methods, case reasoning, decision tree, rule reasoning, fuzzy set, neural network, and genetic algorithms are applied to process the information; and useful analysis information is obtained. Finally, classification analysis, association analysis, cluster analysis, and predictive analysis of relevant data are realized. Therefore, the intelligent manufacturing technology architecture for intelligent CNC machining of low-rigidity parts is shown in Figure 2.

## 3. Research on Key Technologies of Intelligent Manufacturing

### 3.1. Intelligent Decision-Making Based on Deep Learning

Reasonable cutting parameters are very important to improve the cutting efficiency and precision of the machined parts, improve the tool life, and reduce the processing energy consumption. Conventional cutting parameters are optimized with machining cost and efficiency as optimization objectives, using various algorithms. In recent years, some scholars have also considered the impact of energy consumption, cutting force, surface roughness, and other factors on the optimization process. However, these optimization methods cannot correct cutting parameters in real time according to the processing state, which limits their applicability. Most of them cannot achieve real-time guidance of the cutting process and cannot interact with information. Moreover, it is difficult to achieve real-time closed-loop control of complex machining conditions only by using monitoring signals without considering the geometry and process information of the workpiece to be machined [19]. Therefore, the synchronous correlation of geometry, machining process information, and monitoring information is the key to realizing intelligent cutting.

**Figure 3:**
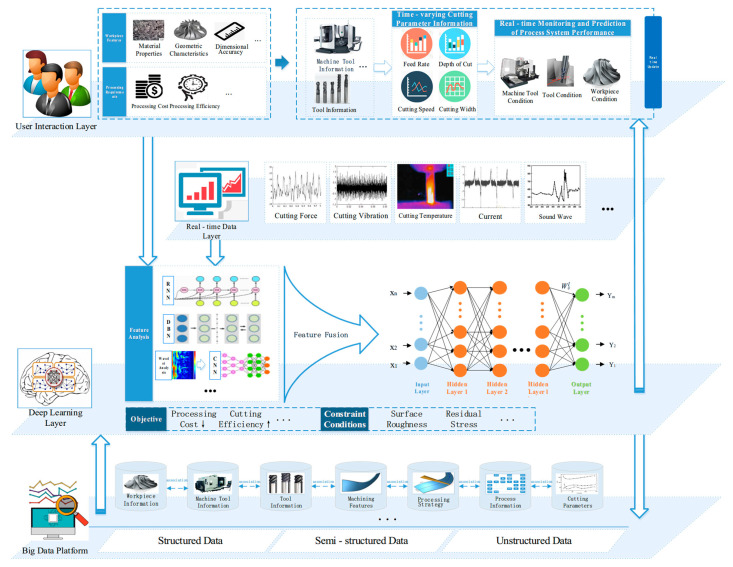
Intelligent decision-making process based on deep learning.

In the machining process of low-rigidity parts, there are very complex state changes in machine tools, workpieces, and tools, which involve machine tool error characteristics, machine tool dynamics characteristics, machine tool energy consumption characteristics, and poor material processing technology. Machining deformation easily occurs under the influence of the cutting force, cutting chatter, and other factors. Conventional mathematical models are difficult to use to accurately simulate the cutting process, control processing accuracy, and improve processing efficiency. Therefore, this paper proposes an intelligent decision-making method based on deep learning to guide cutting machining, as shown in Figure 3.

Supported by the big data platform, intelligent decision making based on deep learning mainly includes three layers: user interaction layer, real-time data layer, and deep learning layer:

(1) User interaction layer

The user interaction layer is mainly used for information exchange with the user and visualization of the cutting process. The information input of the system is transmitted to the deep learning layer through this layer. After model analysis, the basic information, such as the machine tool and tool, is first fed back to the user, and the user can choose the machine tool and tool themselves. After user confirmation, the deep learning layer will be based on the information of the real-time data layer, real-time feed *f*, back cutting amount *a*_p_, cutting width *a_e_*, cutting speed *v*_c,_ and other data through the form of dynamic curves for user visualization. At the same time, through the support of the deep learning layer, users can intuitively see the deformation of the workpiece, the wear of the tool, the energy consumption of the machine tool, etc., to achieve the purpose of monitoring the state of the workpiece, the state of the tool, and the state of the machine tool.

(2) Real-time data layer

The real-time data layer is the data supply layer, and its data sources are two: (1) In the simulation cutting process, the prediction results from the real-time output of the simulation cutting process, including cutting force, cutting temperature, cutting vibration, current, acoustic signal, and other data. (2) In the actual cutting process, the dynamometer, acceleration sensors, thermal imagers, and other sensor equipment from the actual cutting process provide data. Including real-time cutting force information, real-time cutting temperature image, real-time cutting vibration information, real-time current information, real-time acoustic information, etc., provide information sources for the deep learning layer.

(3) Deep learning layer

The deep learning layer is the core of intelligent decision-making. Users put forward the workpiece characteristics and processing requirements in the interaction layer and pass the information to the deep learning layer for feature analysis. With the support of the data service layer, the machine tool intelligent matching is carried out to recommend suitable machine tools, and the tool intelligent matching is carried out to recommend existing suitable tools. Aiming at intelligent tool matching, the geometric features of typical low-rigidity parts in aerospace are represented by multiple engineering drawings containing rich information, which are used as training sets to train algorithm models, such as Deep Residual Network (ResNet). The corresponding relationship between each machining feature and each cutting tool is established to transform tool selection into a feature recognition problem [9]. If the appropriate tool is not matched, or the machining effect of the matched tool is not ideal, the tool-related design information with the highest matching degree is provided for the custom tool design to provide a theoretical basis for the design of the tool used for the next processing of similar structural parts.

The deep learning layer aims at low machining cost, high machining efficiency, long tool life, and low carbon emission, and takes the range of cutting parameters, surface roughness, and residual stress as constraints. Deep learning algorithms such as Restricted Boltzmann Machine (RMB), MultiLayer Perceptron (MLP), Artificial Neural Network (ANN), both short-term and Long Short-Term Memory (LSTM), Convolutional Neural Networks (CNN), Deep Belief Networks (DBN), etc., can be used to train multi-layer feedforward neural network models. These models, or optimization models combining two or more algorithms, can be developed to establish a predictive model in line with the cutting process of low-rigidity parts.

In order to eliminate the structural differences between various types of data, different deep network learning models can be built according to the nature of each signal in the cutting system. For example, a Recursive Neural Network (RNN) model is constructed along LSTM for one-dimensional time series, and a CNN model is proposed for two-dimensional image input. For important monitoring signals, multi-view methods are adopted, such as time series processing by wavelet transform, time-frequency image processing by convolutional neural network, and original time signal processing by DBN, so as to achieve different mode signals to produce different features, and finally, to produce unified features through feature fusion [20]. The model is trained by the cutting history data of low-rigidity parts to make the model suitable for the cutting conditions of low-rigidity parts.

➀ Machine tool state prediction

Machine tool state prediction is the evaluation of the machine tool’s health state. This paper mainly considers the state of the machine tool spindle and energy consumption in the cutting process. By considering the contact characteristics of the tool-jacket-tool shank and tool-spindle joint [21], as well as the mathematical relationship between the machine tool’s dynamic performance and its position and attitude [22], and by using modal analysis to estimate the dynamic modal parameters of the machine tool machining process [23], the signal can analyze real-time characteristic signals such as vibration signal, acoustic emission signal, and current signal for analysis. A comprehensive machine tool state model based on deep learning was established to obtain the performance changes of the machine tool spindle system, that is, the real-time operating state of the spindle system, and the energy consumption of the machine tool, and certain fault diagnosis and fault warning were carried out to obtain the machine tool state during the cutting of low-rigidity parts.

➁ Tool state prediction

Tool state prediction is the evaluation of the tool processing state. According to the monitoring of cutting process signals such as cutting force, vibration signal, and acoustic emission in the cutting process, deep learning algorithms such as multi-scale convolutional long short-term memory model and bidirectional LSTM model are applied to monitor and predict tool wear [24] and damage, respectively, and predict the remaining life of the tool [25] in order to obtain the real-time tool state in the machining process of low-rigidity parts.

➂ Workpiece state prediction

Workpiece state prediction is the assessment of the workpiece state in the cutting process. Deep learning algorithms such as DBN are applied to analyze the cutting force signal, vibration signal [26], cutting heat signal, acoustic emission signal, current signal and other features, combined with the geometric features of low-rigidity parts [27], and directly model the cutting state based on the original measured signals of various types. In order to obtain real-time dynamic cutting parameters, a characteristic environment for monitoring cutting conditions was established. Meanwhile, real-time deformation of low-rigidity parts was predicted to obtain real-time cutting conditions of low-rigidity parts during cutting.

(4) Data service layer

The data service layer is mainly supported by the big data platform and stores the relevant data result information. With the support of the big data platform, the model is trained by the historical data of the cutting of low-rigidity parts to obtain a suitable model for the low-rigidity parts cutting process under working conditions.

After the data processing, calculation, and analysis based on big data, the output results are provided to the cutting simulation part based on the geometric characteristics of the parts to simulate the cutting process of the low-rigidity parts, so as to realize the prediction of the CNC machining process and find the possible problems in the cutting process in time. The system can predict the “burst” condition, improve the machining efficiency of parts and the flexibility of the process system, and provide technical support for the monitoring and control of CNC machining based on state perception. At the same time, all kinds of result information are provided to the user interaction layer for visualization, so that users can intuitively see the changes in the cutting process and output the result information.

The machining accuracy requirements for aeronautical thin-walled or weak-rigidity components are typically stringent due to their critical role in aircraft performance and safety. Generally, the dimensional tolerances for such parts range between ±0.01 mm to ±0.05 mm, while surface roughness requirements often fall within Ra 0.4 μm to Ra 1.6 μm. In order to improve the machining accuracy and surface quality of low-rigidity parts, many scholars at home and abroad have conducted a lot of research on the machining deformation rule and the prediction and compensation of machining errors. Wang et al. [28] proposed a real-time adjustment method of feed speed based on real-time measurement of cutting force, aiming at the problem of easy deformation in cutting of integral multi-frame low-rigidity parts. ZhengZhang [29] studied the effect of material removal on the residual stress of high-strength aluminum alloy parts to reduce processing deformation. Ratchev et al. [30] predicted the machining accuracy errors of low-rigidity parts and proposed error compensation strategies for force, heat, and residual stress deformation. However, in the process of NC machining, the whole structure has time-varying characteristics of stress and stiffness as the material is removed. These off-line or pre-optimization strategies are difficult to guarantee under the current complex and changeable processing conditions and affect the control effect. Considering that the stiffness of low-rigidity parts would change with the process of machining, Guiassa [31] proposed an error compensation method based on the stiffness change. Wang Yongqing [32] studied the real-time compensation of thermal error. Yang and Choi [33] proposed a method based on online cutting force monitoring and calculation of tool deformation to compensate for the tool track in real time.

### 3.2. Custom Tool Design Based on Structural Characteristics

The performance of the machine tool and the realization of the workpiece processing accuracy are closely related to the performance of the tool. With the continuous improvement of the speed of product replacement, especially the low-rigidity parts of the aerospace industry, with many kinds, small batches, complex structures, and even special-shaped structure, the current batch tool production mode gradually cannot adapt to the current development trend of the aerospace industry [34,35,36]. Therefore, the low-rigidity parts of aerospace put forward the requirements of high precision, high efficiency, high reliability, and specialization. Therefore, the “specialization” of the tool is an important direction to improve the performance of the tool and meet the processing needs of low-rigidity parts. The improvement of the degree of specialization will inevitably lead to an increase in the type of tool and a reduction in the batch, so it is of great significance to study the precision, flexibility, and adaptability of the tool design and manufacturing.

**Figure 4:**
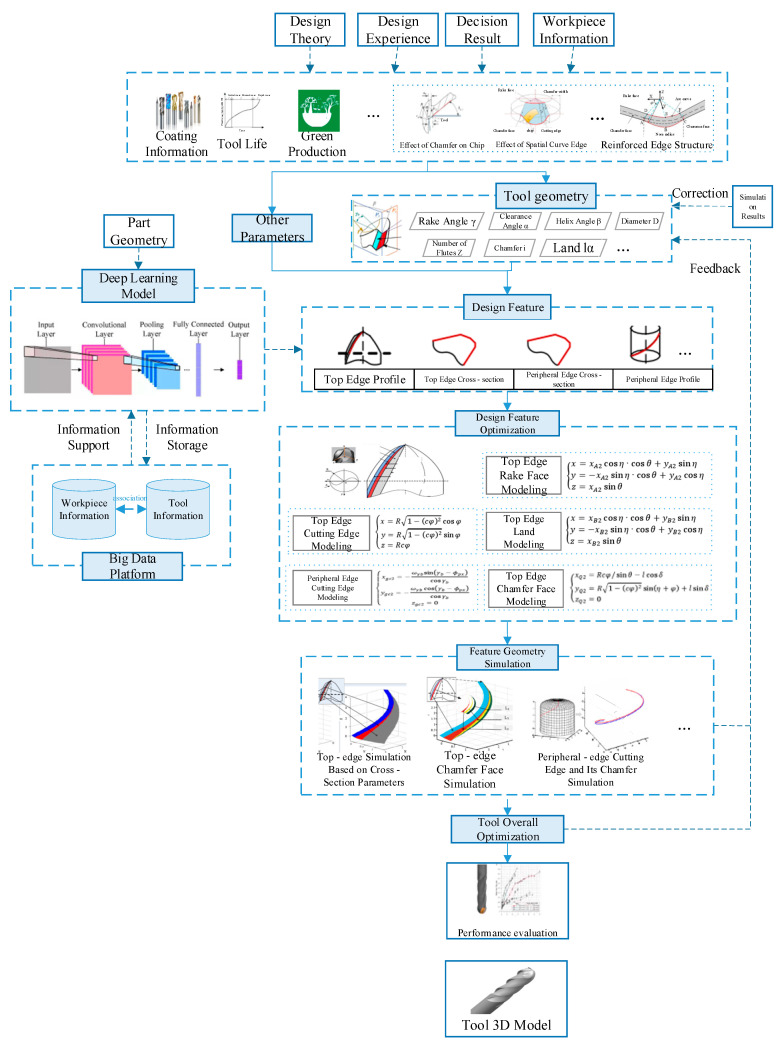
Design process of customized tool based on structural features.

At present, in the tool industry, tool design methods mainly include the tool design based on cutting theory, the tool design method based on experience and experiment, and the computer-aided tool design method. The tool design method based on theory is to design the parameters of the cutting edge and geometrical angle according to metal cutting theory and the parametric equation of a regular profile. The tool design method based on experience and experiment is mainly based on the relevant theories of tool angle and tool material in metal cutting, as well as the basic laws and experiences of cutting force, cutting heat, tool wear, tool damage and tool life, and the initial selection of tool materials and tool geometric parameters for one or several objectives. A large number of tool material selection tests and tool geometric parameter optimization tests are carried out, and the optimal tool parameters are selected by analyzing the test results. Computer-aided tool design is the application of computer technology, database technology and software in tool design, through the establishment of mathematical model or finite element model to express the geometric characteristics of the tool, with the help of three-dimensional modeling software, to design a reasonable and effective blade structure, in order to improve the efficiency and level of tool design. It can be seen that the current research on the intelligent tool design process is still relatively few. Therefore, according to the structural characteristics of the tool used in the material processing of typical aerospace parts, this paper proposes the design process of the customized tool based on the structural characteristics, as shown in Figure 4.

With the information support of the big data platform, the design of customized tools based on structural characteristics should not only consider the conventional tool design, but also consider the influence of the design theory and design experience of the tool, coating information, tool life, green resource utilization and other factors, but also for the low-rigidity parts are mostly difficult to process materials such as titanium alloy and easy to deform. The effect of chamfering and space curve edge on chips and the strengthening of edge structure [37] are considered to reduce tool wear and damage and improve tool life.

At the same time, the intelligent decision part also provides the algorithm decision and information support for the tool design process. The deep learning part provides a special sub-module for customized tool design based on structural features. It uses historical data to represent the geometric features of typical low-rigidity parts and tools in aerospace through multiple engineering drawings containing rich information, and uses them as a training set to train the algorithm model and establish the corresponding relationship between the geometric features of each part and the geometric features of the tool. This greatly improves the efficiency of tool design and provides a design reference for the specialized design of tools.

By analyzing the contact conditions between the tool and the free-form surface, the relationship between the tool parameters and the curvature of the surface is derived, and the relationship between the geometry of the part and the geometry of the tool is obtained [38]. Thus, this more accurately determines the basic parameters of the tool geometry, including front Angle *γ*, back Angle *α*, helix Angle *β*, diameter *D*, edge number *Z*, chamfering *l*, edge band *l*_α_, etc. Based on these, design characteristics like top edge type, top edge section, edge section, and edge profile can be modeled and optimized to achieve the tool structure characteristics and low-rigidity parts characteristics. Using Matlab and other software based on the section parameters of the top edge simulation, top edge chamfering surface simulation, edge cutting edge and chamfering simulation, and other characteristic geometric simulations, it is possible to achieve the overall optimization of the tool. If there is any problem in the process of simulation optimization, return to the initial stage of design and change the tool geometry parameters. Finally, the shape, performance, and use of the tool are linked [39] to achieve special tool design for parts processing requirements. Thus, customized design of specialized cutting tools for low-rigidity parts can be obtained, providing information support for the simulation part.

### 3.3. Machining Simulation Based on Geometric Features of Parts

**Figure 5:**
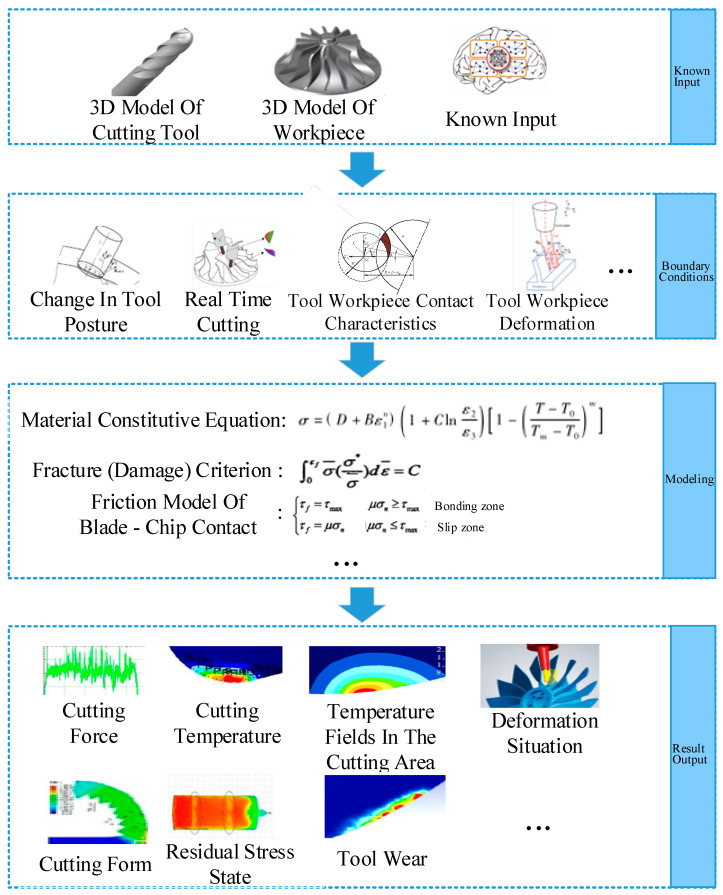
Machining simulation process of low-rigidity parts.

In the field of metal cutting, using finite element simulation software to predict the cutting force, cutting temperature, workpiece deformation, chip shape, and machining quality in the process of cutting has become a very effective means. However, the typical low-rigidity parts of aerospace and aviation have high precision requirements and are all thin-walled parts with complex curved surfaces. In the cutting process, deformation and vibration are large, and the simulation theoretical model adopted in the conventional cutting process cannot achieve the expected simulation effect. Therefore, the simulation process for the cutting of low-rigidity parts is proposed in this paper, as shown in Figure 5.

In the machining simulation process of low-rigidity parts, finite element simulation is carried out according to the data information provided by the 3D model of parts, the 3D model of tools, and the intelligent decision. In this part, according to the material characteristics and structural geometric characteristics of typical aerospace thin-wall parts, considering the change of tool attitude, time-varying cutting and tool workpiece contact characteristics, for time-varying cutting, finite element software can be used to simulate the cutting process, which can obtain the relationship between the time stress and the change of the tool stress. Due to the large deformation in the cutting process of low-rigidity parts, the feedback effect of tool workpiece deformation field on heat source heating should be emphasized in the simulation process [40], so as to re-establish the appropriate material constitutive equation, reconstruct the cutting fracture criterion, and obtain the tool chip contact friction model according to the structural characteristics of parts. Establish a 3D cutting simulation model with a continuous adaptive mesh method without losing key information [41]. Thus, precise simulation of cutting forces, cutting heat, stress and temperature fields, tool wear, and chip morphology during the machining process of typical thin-walled aerospace parts can be achieved to understand the cutting state of the tool. At the same time, the thin walls of low-rigidity parts are sensitive to the residual stress, and the deformation is obvious during machining, so the residual stress state and the deformation of the workpiece tool should be simulated to realize the visualization of the cutting simulation process.

**Figure 6:**
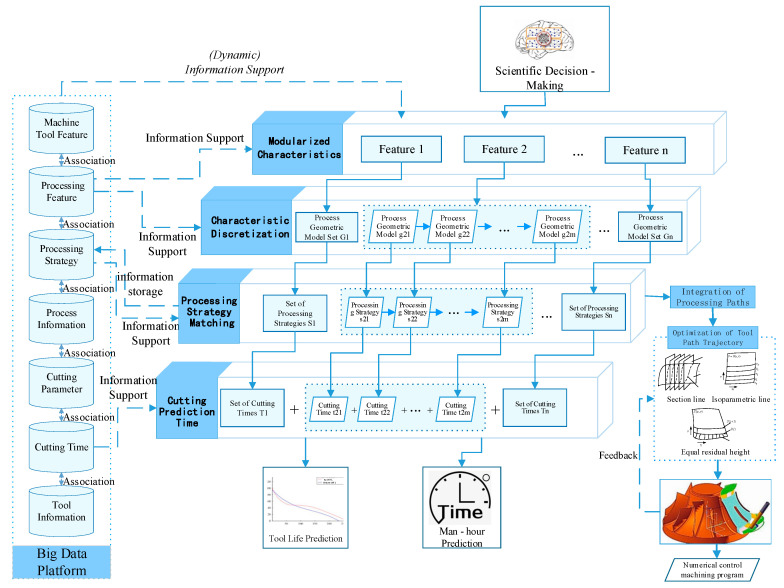
Intelligent process planning process based on geometric features of parts.

Low-rigidity parts have thin walls and complex structures, so they are prone to bumps and interference in manufacturing. Therefore, tool path planning is an important link in the machining process of low-rigidity parts. The optimized tool path can significantly improve the machining efficiency of curved surfaces, and the machining cycle of low-rigidity parts is long, which is also crucial to the prediction of machining hours and tool life. Machining features are an effective means to realize efficient and high-quality CNC programming of complex structural parts, but the same type of machining features are only similar in geometric shape and processing technology, and are not exactly the same. Based on the above research, this paper takes the geometric features of parts as the research object and proposes an intelligent process planning based on the features of parts, as shown in Figure 6.

In the process of feature-based intelligent process planning, the tool path in actual machining is composed of discrete tool position data in motion order, so the tool path can be regarded as a collection of tool position data. The process includes feature modularization, feature discretization, machining strategy matching, machining path integration, and machining tool path optimization, and finally, the NC machining program is automatically obtained. At the same time, it can also predict the cutting time and the remaining life of the tool, and obtain the processing time of the part. The big data platform provides data support for the realization of the intelligent process planning process, including machine tool characteristics, machining characteristics, machining decisions, process information, cutting parameters, cutting time, and tool information.

Machine tool features and intelligent decision results provide information sources for the feature-based intelligent process planning process. Firstly, the geometric features of parts are modularized according to the big data platform. Obtain *n* features: Feature 1, Feature 2. Characteristic *n*; Under the support of the existing processing feature database, each feature is discretized, and the process geometric model set *G_n_* corresponding to the feature is obtained. The set includes *m* process geometric models *g_n1_*, *g_n2_*, …, *g_nm_*. With the support of the machining strategy library, each process geometric model *g_nm_* has a corresponding machining strategy *s_nm_*, so as to obtain the machining strategy set *S_n_* for each feature, and finally obtain the machining strategy set of the part, complete the strategy storage, and expand the machining strategy library. Then, through the integration of machining paths, the tool path optimization methods including cross-section line method, isoparametric line method and equal residual height method are adopted to obtained the optimized tool path trajectory, and the final machining results are fed back to the tool path optimization method, and a complete set of CNC machining program will be obtained after the machining is completed. The cutting time provided by the big data platform provides information support for the cutting time prediction in CNC machining. Through the above process route analysis, the tool can probabilistically forecast and estimate working hours. For example, Liu Changqing et al. [42] extracted the influencing factors of working hours based on the processing characteristics by analyzing the structural characteristics and process characteristics of aircraft structural parts. A feature-based two-level structure working hour prediction model is proposed to solve the problem that the prediction method of NC machining working hours cannot take into account both accuracy and efficiency, so as to obtain the intelligent process planning based on the geometric structure features of parts.

At the same time, with the support of intelligent decision-making, the workpiece should be simulated cutting before the actual processing, and the data generated in the simulation processing process is processed and analyzed. In order to achieve efficient and accurate cutting, the machining error should be predicted. For the machining process prediction and online compensation, for example, by considering the stiffness changes of low-rigidity parts at different stages [31] and the machine tool space error [43], etc., the optimal spatial error model, which comprehensively reflects geometric error, thermal error, and cutting force error, is established to predict the mechanical deformation, thermal deformation and residual stress deformation of low-rigidity parts in simulation analysis.

### 3.4. CNC Machining Monitoring and Control Based on State Perception

**Figure 7:**
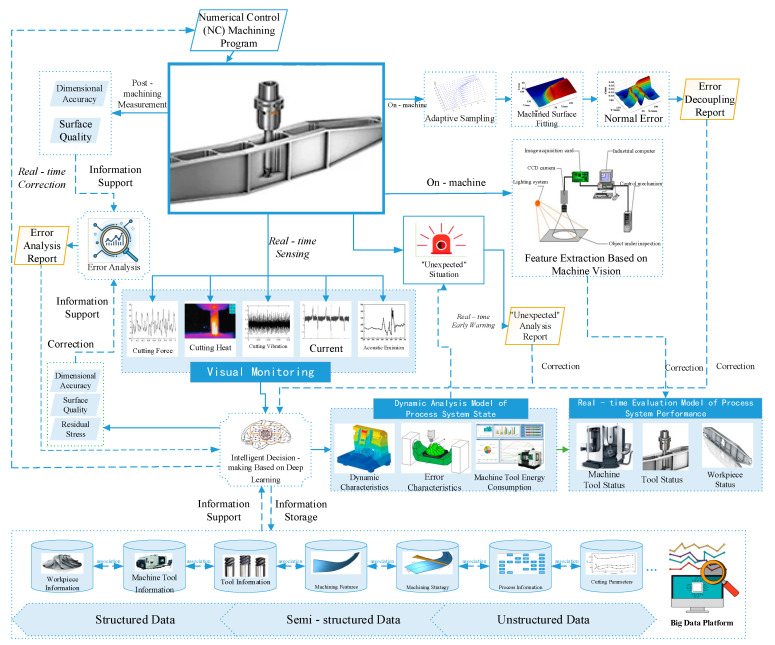
Monitoring and control process of NC machining based on state perception.

The machining cycle of low-rigidity parts is long, the processing is prone to deformation, and the processing quality is difficult to control. In order to meet the productivity and accuracy requirements, the cutting process of such parts needs real-time monitoring and adaptive control under changing working conditions. The machining quality of the parts is affected by complex factors such as weak stiffness characteristics, residual stress, excessive cutting force, clamping force, processing sequence, etc., making the machining accuracy difficult to control. At the same time, it is also affected by the action mechanism, working performance, regular characteristics, and realization methods of the workpiece blank and fixture system, milling tool and tool handle system, machine tool body and drive system, CNC system, etc. Especially for the complex parts widely used in the aerospace manufacturing industry, the removal of workpiece materials causes the machine tool, workpiece, and tool to undergo very complex state changes. These include complex tool-cutting mechanism characteristics, machine tool multi-axis linkage, dynamics–kinematics–process system error–energy consumption, and other characteristics. With the support of big data information, the intelligent CNC system construction and system integration are put forward high requirements and implementation difficulties. Based on the above research, this paper proposes the monitoring and control process of CNC machining based on state awareness, as shown in Figure 7.

CNC machining monitoring and control based on state perception is realized with the support of a big data platform. In the cutting process of low-rigidity parts, cutting force, cutting vibration, cutting heat, current, and acoustic emission are measured by sensors such as a dynamometer, an acceleration sensor, and a thermal imager. For these multi-source heterogeneous data, it is difficult to collect and process them by a single communication protocol. The communication architecture and access strategy based on OPCUA technology and MTConnect protocol can be used to collect them [20], and then the data can be transformed and analyzed to achieve visual monitoring. It can then pass the information to intelligent decision-making based on deep learning and make intelligent decisions on real-time data with the support of a big data platform. It is necessary to achieve equipment operation visualization, equipment alarm information visualization, remote diagnosis visualization, networking status monitoring visualization, equipment fault maintenance history visualization, and equipment file visualization, to ensure that the hardware part in the cutting process can operate normally. At the same time, the intelligent decision-making part conducts dynamic analysis of the state of the process system, including dynamic characteristic analysis, error characteristic analysis and machine tool energy consumption analysis, according to the real-time cutting information of the machining process and processed by the deep learning model, so that users can monitor the state and energy consumption of the spindle system (machine tool state) in real time. Tool wear, remaining life, and expected tool change time (tool state), workpiece stress and deformation, surface integrity (workpiece state), achieve real-time visualization of machine tool, tool, and workpiece state. Through the real-time monitoring and visualization of the cutting process, the “unexpected” conditions in some cutting processes can be given a real-time early warning, and the analysis report that may lead to “unexpected” conditions can be output, so as to modify the intelligent decision model. At the same time, intelligent decision-making can modify the numerical control program in real time according to the monitoring results to achieve accurate numerical control machining.

The monitoring and control of NC machining based on state awareness can obtain the tool state by using the machine vision-based method in machine form. For example, after the tool surface image is collected, the background noise is removed by a morphological method, the tool wear area is enhanced, the tool condition is identified, and the wear degree of the tool is quantified [44], thus obtaining the actual tool condition. Compared with the predicted results, the evaluation model of intelligent decision is modified to improve the accuracy of the model. In the same way, the parts can be adaptively sampled by means of in-machine measurement, and the machining surface can be fitted to obtain the normal error after data processing, so as to obtain the error data during the machining process. The error decoupling report can be obtained by comparing the workpiece requirements and simulation results, and the error data can be fed back to the intelligent decision module to correct the deep learning algorithm model. Finally, after the parts are processed, the dimensional accuracy and surface accuracy can be measured, and the error decoupling report can be obtained by comparing with the predicted value and analyzing the processing error, so as to modify the intelligent decision model. Finally, the cutting parameters can be automatically optimized according to the real-time working conditions, and the processing state can be adjusted to achieve the purpose of high-precision and efficient intelligent adaptive cutting.

### 3.5. Case Analysis: Precision Machining of Titanium Alloy Engine Blade Tenon

Case analysis serves as validation for the proposed methodology, demonstrating its operational efficacy in realistic scenarios [45]. The following is a case design of parts processing for intelligent manufacturing, covering key technical links such as deep learning decision-making, tool design, simulation, and monitoring.

#### 3.5.1. Part Overview and Challenges

The selected aerospace component is a titanium alloy (Ti-6Al-4V) engine blade root, requiring ultra-tight tolerances (±5 μm) and a superior surface finish (Ra < 0.8 μm). Traditional machining of Ti-6Al-4V faces significant challenges, including rapid tool wear, poor thermal conductivity, and work hardening, leading to high scrap rates (>15%) and inefficient production cycles.

#### 3.5.2. Intelligent Machining Workflow

(1) Deep learning-based process optimization

A multi-modal neural network integrates historical machining data (cutting forces, vibration signals, tool wear), material microstructure (SEM/EBSD grain orientation), and 3D point cloud scans of raw stock to optimize process parameters. The model architecture combines a CNN for image processing, an LSTM for time-series sensor data, and a Transformer-based fusion layer to generate real-time recommendations for spindle speed, feed rate, and depth of cut. Key outputs include:

➀ Dynamic parameter adjustments (e.g., optimal vc = 50 m/min, fz = 0.08 mm/z, ap = 0.3 mm).
➁ Tool remaining useful life (RUL) prediction with <5% error.

(2) Geometry-Driven Tool Design Methodology

The cutting tool is custom-designed based on the blade root’s geometric features, including:

➀ Complex 3D profiles (dovetail grooves, concave/convex surfaces).
➁ High aspect ratio (deep, narrow slots requiring long-reach tools).
➂ Tight corner radii (R0.2–0.5 mm, demanding micro-grain carbide tools).

Design workflow:

➀ CAD Model Analysis–Extract critical features (e.g., undercuts, draft angles) from the blade root’s STEP file.
➁ Cutting Engagement Simulation–Identify high-stress zones using CAM software (e.g., Siemens NX).
➂ Tool Geometry Optimization–Adjust rake angle, helix angle, and edge preparation based on localized forces.

(3) Cutting process simulation via Digital Twin

A multi-physics digital twin combines Discrete Element Method (DEM) for chip formation and Finite Element Method (FEM) for thermal-stress analysis. The simulation predicts:

➀ Cutting temperature distribution (error < 8% vs. IR thermal imaging).
➁ Chatter frequency thresholds (92% detection accuracy).

The model is calibrated using AdvantEdge FEM software with real machining data.

(4) Real-time monitoring and adaptive control

The DMG Ultrasonic 20 linear machining center is equipped with:

➀ Acoustic emission sensors (1 MHz sampling rate) for micro-fracture detection
➁ High-resolution industrial cameras (5 μm/pixel) for in-process surface roughness measurement

A closed-loop control system dynamically adjusts parameters:

➀ Chatter suppression: Reduces spindle speed by 10% if vibration exceeds 0.5 g.
➁ Surface quality compensation: Triggers finish passes if Ra > 0.8 μm.

This case study demonstrates how smart manufacturing technologies—deep learning-driven optimization, geometry-driven tools, digital twin simulation, and real-time adaptive control—can significantly enhance the machining of aerospace components. The framework is scalable to other critical parts (e.g., turbine disks, compressor casings) and highlights the Industry 4.0 transition in high-value manufacturing.

## 4. Conclusions

Aiming at the characteristics of typical parts used in aerospace engines, such as difficult machining, complex structure, and weak rigidity, this paper studies the modeling, simulation, and prediction of the machining process, as well as the monitoring and control of the machining system, with the help of advanced monitoring and simulation methods. It proposes an intelligent manufacturing technology framework for machining. The conclusions are as follows:

(1) The cutting processing mode proposed in this paper consists of four parts: intelligent decision making based on deep learning, custom tool design based on structural features, cutting simulation based on geometric features of parts, and CNC machining monitoring and control based on state perception, which realizes the intelligent cutting closed-loop system.
(2) The system with the cutting processing mode can monitor the key signals of the cutting process in real time. According to the set evaluation criteria, it conducts calculation, analysis, diagnosis, optimization, and decision-making to realize the real-time monitoring, intelligent optimization, and adaptive control of the cutting process of low-rigidity parts.
(3) The cutting processing mode continuously enriches the knowledge base in the cutting process, has a certain self-learning ability, can analyze, judge, and plan its own behavior, and promotes the effective operation of the digital production process. Finally, the feasibility of the proposed processing mode was verified through cases.

This paper only explains and explores the proposed intelligent manufacturing technology architecture and its key technology system from the perspective of the realization idea. In the future, on the basis of intelligent manufacturing technology, through digital twin technology, Internet of Things technology and cloud computing technology, the relevant equipment will be intelligently interconnected to achieve wireless communication, real-time perception, online monitoring, simulation, intelligent optimization, autonomous decision-making, accurate control and rapid execution of the cutting process of typical low-rigidity parts of aerospace engines.

## Data Availability

The datasets used or analyzed during the current study are available from the corresponding author on reasonable request.
